# DB‐1419: Augmenting the Translational Potential of Preclinical Findings for Implementation in the Clinic

**DOI:** 10.1111/jcmm.70784

**Published:** 2025-08-05

**Authors:** Kostas A. Papavassiliou, Kyriaki Cholidou, Athanasios G. Papavassiliou

**Affiliations:** ^1^ First University Department of Respiratory Medicine, ‘Sotiria’ Chest Hospital, Medical School National and Kapodistrian University of Athens Athens Greece; ^2^ Department of Biological Chemistry, Medical School National and Kapodistrian University of Athens Athens Greece

**Keywords:** antibody‐drug conjugate, checkpoint blockade, DB‐1419, targeted therapy

A recent study published by Li and colleagues in *Clinical Cancer Research* preclinically evaluates DB‐1419, a bispecific antibody‐drug conjugate (bsADC) targeting programmed death‐ligand 1 (PD‐L1) and B7‐H3 (also known as cluster of differentiation 276 (CD276); a member of the B7 ligand family of immune checkpoint molecules), conjugated to a proprietary topoisomerase I inhibitor (P1003) [1]. The therapeutic rationale of combining immunotherapy with a high drug‐to‐antibody ratio (DAR) chemotherapeutic construct is ambitious, timely and relevant [2]. However, we wish to highlight several conceptual and methodological limitations that, if addressed, could enhance the clarity, reliability and translational value of the findings.

Initially, the reported weak correlation between antigen binding and cytotoxicity (*R* = −0.2) raises concerns about the dependency of DB‐1419's efficacy on PD‐L1/B7‐H3 expression. Although the investigators speculate that this may reflect payload‐driven or bystander effects, a mechanistic distinction would be essential to inform clinical trial design and patient selection strategies. Future research must unravel these effects, potentially via clustered regularly interspaced short palindromic repeats (CRISPR) knockout models, quantitative immunohistochemistry (qIHC) or positron emission tomography (PET)‐based target engagement, to develop reliable predictive biomarkers for clinical stratification.

Next, while the study reports lysosomal trafficking and payload release, the internalisation kinetics and relative contributions of each target remain inadequately characterised. Moreover, the high DAR (~8) may increase potency but could adversely affect pharmacokinetics, aggregation and off‐target effects—risks that are insufficiently addressed. Competitive binding assays or internalisation‐blocking studies would elucidate the contribution of each target and inform future ADC optimisation.

Furthermore, the dual immunomodulatory functions of DB‐1419, namely, PD‐L1 blockade and antibody‐dependent cellular cytotoxicity (ADCC), are key to its design but are not supported by rigorous in vivo immune profiling. The absence of comprehensive immunophenotyping (e.g., T cell activation, dendritic cell maturation, tumour microenvironment (TME) analysis or cytokine profiling within the tumour) limits our understanding of how DB‐1419 modulates the tumour immune landscape. Future work employing flow cytometry (FCM), multiplex IHC (mIHC; the simultaneous detection of multiple markers on a single tissue section) or single‐cell RNA sequencing (scRNA‐seq) would help in clarifying the immunologic underpinnings of DB‐1419's efficacy.

Other limitations also warrant closer scrutiny. The in vivo models used lack the complexity of the human TME and may overestimate immunologic effects. Orthotopic or syngeneic humanised models would better capture spatial heterogeneity and immune suppression; hence, significantly improve the translational confidence.

Additionally, while the authors demonstrate preclinical safety in cynomolgus monkeys, the findings of pulmonary inflammation, thymic lymphocyte (T lymphocyte) depletion and liver enzyme elevation deserve further exploration. Given the known low‐level expression of PD‐L1 and B7‐H3 in normal human tissues, especially the thymus, lung and placenta, detailed safety studies using human primary cells or organoids would be prudent prior to broader clinical translation.

Lastly, no comparative benchmarking against clinically advanced ADCs was presented, limiting insight into DB‐1419's relative value. A head‐to‐head comparison in terms of pharmacokinetics, toxicity, immunogenicity and efficacy would contextualise these findings more effectively and better support the argument for the advancement of DB‐1419.

In summary, DB‐1419 represents a potentially transformative approach by merging dual immune checkpoint blockade with a cytotoxic payload; yet addressing the above critical issues/limitations would substantially strengthen the applicability of the findings by Li et al. in the clinical setting [3, 4].

## Author Contributions

**Kostas A. Papavassiliou:** conceptualisation (lead), data curation (lead), writing – original draft (lead). **Kyriaki Cholidou:** data curation (equal), writing – original draft (equal). **Athanasios G. Papavassiliou:** conceptualisation (lead), data curation (lead), supervision (lead), writing – review and editing (lead).

## Conflicts of Interest

The authors declare no conflicts of interest.

## Data Availability

Data sharing not applicable to this article as no datasets were generated or analysed during the current study.
